# The Bumpy Road towards mTOR Inhibition in Glioblastoma: Quo Vadis?

**DOI:** 10.3390/biomedicines9121809

**Published:** 2021-12-01

**Authors:** Kostas A. Papavassiliou, Athanasios G. Papavassiliou

**Affiliations:** Department of Biological Chemistry, Medical School, National and Kapodistrian University of Athens, 11527 Athens, Greece; konpapav@med.uoa.gr

**Keywords:** glioblastoma multiforme, mTORC1, mTORC2, mTOR inhibitors, mTOR signaling

## Abstract

Glioblastoma multiforme (GBM), a grade IV astrocytoma, is a lethal brain tumor with a poor prognosis. Despite recent advances in the molecular biology of GBM, neuro-oncologists have very limited treatment options available to improve the survival of GBM patients. A prominent signaling pathway implicated in GBM pathogenesis is that of the mechanistic target of rapamycin (mTOR). Attempts to target the mTOR pathway with first-generation mTOR inhibitors appeared promising in the preclinical stage; however, results have been disappointing in clinical trials, owing to the heterogeneous nature of GBM, escape mechanisms against treatment, the blood–brain barrier, drug-related toxicities, and the imperfect design of clinical trials, among others. The development of next-generation mTOR inhibitors and their current evaluation in clinical trials have sparked new hope to realize the clinical potential of mTOR inhibitors in GBM. Meanwhile, studies are continuously furthering our understanding of mTOR signaling dysregulation, its downstream effects, and interplay with other signaling pathways in GBM tumors. Therefore, it remains to be seen whether targeting mTOR in GBM will eventually prove to be fruitful or futile.

## 1. Introduction

Multiple oncogenic signaling pathways are known to be deregulated in glioblastoma multiforme (GBM), contributing to its pathogenesis. Among the signaling pathways that have been pharmacologically targeted in GBM is the mechanistic target of the rapamycin (mTOR) pathway. Although drugs modulating this pathway showed promising results in preclinical studies, their application in clinical trials proved to be disappointing, as a result of GBM heterogeneity, poor pharmacology, inherent and acquired resistance to mTOR inhibitors, and molecularly unselected cohorts of GBM patients in these clinical trials (Table 1) [1,2,3]. The failure of mTOR inhibitors to demonstrate clinical benefit until now does not mean that this therapeutic strategy should be discarded, but rather raises several important issues that need to be addressed in order to reach a verdict regarding the future of such agents in GBM management: (a) further research is required to uncover the intricacies of mTOR signaling in GBM, (b) preclinical studies assessing the therapeutic potential of mTOR inhibitors should represent, as close as possible, the conditions of the GBM tumor microenvironment, (c) rational subgroup selection of GBM patients in clinical trials based on the molecular profile of their tumors, and (d) combining mTOR inhibitors with other targeted agents.

## 2. Deconstructing mTOR Biology in GBM

A deep understanding of the molecular circuitry of mTOR signaling in GBM is crucial in order to improve the clinical outcome of mTOR inhibitors. Research efforts have already provided a great amount of information on the role of the mTOR pathway in GBM pathogenesis [4,5]. Compared to mTORC1, less is known about mTORC2; however, the latter is emerging as a significant player in GBM, contributing to numerous oncogenic processes [6,7,8]. New data regarding the two complexes are helping to construct a more detailed picture of mTOR biology in GBM. For example, a recent study revealed that GBM stem cells (GSCs) can activate the mTOR pathway in GBM-associated microglia, resulting in an immunosuppressive microenvironment that promotes GBM growth [9]. As the authors state, these findings suggest that mTOR inhibitors could also act on microglia to suppress their tumor-promoting effects. Another recent study discovered that both mTORC1 and mTORC2, acting downstream of oncogenic epidermal growth factor receptor (EGFR) signaling, are cooperatively involved in the epigenetic regulation of GBM progression. More specifically, mTORC1 was shown to increase the protein levels of the enhancer of zeste homolog 2 (EZH2), a histone methyltransferase that constitutes the catalytic subunit of polycomb repressive complex 2 (PRC2) and catalyzes the tri-methylation of histone H3 at lysine 27 (H3K27me3), whereas mTORC2 functions to upregulate the intracellular concentration of S-adenosylmethionine (SAM), the substrate for histone methylation. The coordinated activity of both mTOR complexes was demonstrated to promote H3K27me3 and drive GBM growth in vitro and in vivo [10]. Furthermore, mTORC2 functions as an epigenetic regulator of iron metabolism to promote GBM cell survival [11]. Knowledge of the complexity of mTOR signaling in GBM, including regulatory mechanisms and molecular cross-talks with other pathways, is increasingly growing. For example, there is crosstalk between PI3K/mTOR and MEK/ERK signaling that drives the self-renewal maintenance and tumorigenicity of GBM stem cells (GSCs) [12]. Evidence also suggests an interaction between mTORC2 and Hippo signaling that leads to the promotion of GBM growth and invasiveness [13,14]. Concerning regulatory mechanisms, mTORC2 is implicated in a feed-forward loop, involving Akt, heat-shock transcription factor 1 (HSF1), human antigen R (HuR), and Rictor, which enhances mTORC2 activity and GBM growth [15]. All these aspects of mTOR signaling have opened new avenues for research in GBM biology and have provided opportunities for targeting the mTOR pathway through different strategies.

## 3. Novel mTOR Inhibitors

First-generation mTOR inhibitors include rapamycin and chemical compounds derived from rapamycin (rapalogs) that act to specifically block the mTORC1 complex via binding to FK506 binding protein 12 (FKBP12) [16]. Several aspects related to the molecular architecture of mTOR signaling have been found to be responsible for its incomplete suppression by first-generation mTOR inhibitors and the failure of the latter in GBM clinical trials. These include disinhibition of mTORC1-mediated negative feedback loops by rapamycin, which results in the activation of protein kinase B (Akt), eukaryotic translation initiation factor 4E (eIF4E), and mitogen-activated protein kinase (MAPK) survival pathways [17,18,19]; the presence of mTORC1 kinase-dependent activity that is resistant to rapamycin [20]; and the insensitivity of mTORC2 to acute treatment with rapamycin, which allows Akt to be activated [21,22]. The limited capacity of first-generation mTOR inhibitors led to the development of second-generation mTOR inhibitors that bind to the ATP-binding pocket in the mTOR kinase domain of both mTORC1 and mTORC2 and repress their activity. Second-generation mTOR inhibitors include dual phosphatidylinositol-3 kinase (PI3K)/mTOR inhibitors (e.g., PI-103, GNE-477, NVP-BEZ235, BGT226, XL765, SF-1126, and WJD008) and mTORC1/2 inhibitors (e.g., Torin1, Torin2, PP242, PP30, Ku-0063794, WAY-600, WYE-687, WYE-354, INK128, AZD8055, AZD2014, and OSI-027) [23]. Many second-generation mTOR inhibitors have now progressed to GBM clinical trials, and the results of these trials are eagerly awaited. The inevitable emergence of resistance mechanisms to first- and second-generation mTOR inhibitors has fueled research efforts to develop third-generation mTOR inhibitors: bivalent compounds that link rapamycin to second-generation mTOR inhibitors [24]. RapaLink-1, a third-generation mTOR inhibitor capable of crossing the blood–brain barrier, was shown to be more potent in in vitro and in vivo GBM models than earlier generation mTOR inhibitors [25]. Another promising class of mTOR inhibitors that is still in the early phase of drug development is selective mTORC2 inhibitors. The rationale behind the development of such compounds is that the selective blockade of only mTORC2 will not interfere with mTORC1-dependent negative feedback loops and, thus, may be more effective in inhibiting tumor growth [26]. A study identified CID613034 and its analog JR-AB2-011, small molecule inhibitors that are highly selective for mTORC2, which displayed potent anti-tumor effects in GBM cell lines and xenografts, respectively [27]. These novel mTOR inhibitors bring mTOR inhibition in GBM back to the spotlight and offer hope for future GBM clinical trials (Table 2).

## 4. Biologically Relevant Preclinical GBM Studies Accessing mTOR Inhibitors

Ideally, preclinical studies should use in vitro and in vivo models that reflect, as much as possible, the biology of GBM tumors in order to evaluate the effect of targeted drugs and proceed to clinical trials. A recent study that highlights the significance of this concept used the mTORC1/2 inhibitors Torin2, INK-128, and NVP-BEZ235 to investigate their effects on GBM metabolism. Interestingly, the authors discovered that under hypoxic and nutrient-poor conditions, mTORC1/2 inhibitors—much like rapamycin and rapalogs—protected GBM cells by increasing their tolerance to nutrient and oxygen deprivation [28]. GBM tumors are primarily characterized by a nutrient-deficient hypoxic microenvironment; hence, mTOR inhibitors should be used cautiously in GBM patients. This study also hints at the following ideas: (a) combining mTOR inhibitors with other therapies that block the pro-survival effects induced by the former or (b) developing selective mTOR inhibitors that only promote GBM growth inhibition without eliciting a protective response against hypoxia and nutrient deficiency.

## 5. Designing Appropriate GBM Clinical Trials for mTOR Inhibitors

A major drawback of the clinical trials that failed so far is the inclusion of GBM patients without molecularly defining their tumors in terms of mTOR signaling activity. If the clinical outcomes of mTOR inhibitors for GBM are to be improved, the identification and incorporation of predictive biomarkers that can define patients who are most likely to show a response to mTOR inhibitors in clinical trials is of utmost importance. A paradigm of such a molecularly informed clinical trial is that of the novel German N^2^M^2^ trial, which evaluates molecularly matched targeted therapies for newly diagnosed unmethylated isocitrate dehydrogenase (IDH) wild-type GBM patients. Specifically, one of the goals of this trial is to molecularly analyze patient tumor samples, identify and select those patients whose tumors exhibit activated mTOR (mTOR phosphorylated at Ser2448), and subsequently administer temsirolimus along with standard radiotherapy to this particular patient subgroup [29]. Nevertheless, the fact that patients’ brain tumors harbor particular molecular alterations that are used as potential predictive biomarkers does not automatically translate into a sensitivity to drugs targeting these alterations because GBM tumors present remarkable spatial and temporal heterogeneity.

Phase 0 and window of opportunity clinical trials are also a step forward toward accelerating the development of effective mTOR inhibitors and other drugs for GBM; however, it is not without its challenges, given the obstacle of delivering drugs to the central nervous system due to the blood–brain barrier and the difficulty of acquiring brain tumor biopsies [30]. Such studies, if their clinical protocols are standardized, can rapidly assess the pharmacokinetic and pharmacodynamic properties of mTOR-targeting compounds on a patient’s tumors. Therefore, if a compound demonstrates poor pharmacology early on—for example, not being able to penetrate the blood–brain barrier—its further development can immediately be halted.

Obtaining brain tumor tissues from GBM patients at relevant time points during treatment in clinical trials to assess the adequacy of target inhibition is difficult, but it can provide vital information about the biological effects of targeted drugs on patient-derived GBM cells as well as the molecular mechanisms of resistance to targeted therapy. Despite the challenges, a Phase I trial successfully applied this strategy to evaluate the antitumor activity of rapamycin in patients with recurrent phosphatase and tensin homolog (PTEN)-deficient GBM [31]. Similar trials in the future will help pave the way toward developing effective mTOR inhibitors.

Another innovation that will assist in the improvement of the clinical outcome of mTOR inhibitors is the development of noninvasive methods that are able to directly or indirectly assess whether these drugs are, in fact, hitting their intended target. For example, a recent study identified 2-hydroxyglutarate (2HG), via magnetic resonance spectroscopy, as a metabolic biomarker of IDH-mutant GBM response to the dual PI3K/mTOR inhibitor XL765. Additionally, the decreased 2HG levels detected after treatment with XL765 were correlated with improved survival in a GBM mouse model [32]. Further preclinical and clinical studies are required to validate this biomarker.

## 6. mTOR Inhibition in Combination with Other Targeted Therapies

GBM is not a single-pathway disease. Either due to intrinsic multiple oncogenic pathways or the activation of cross-talk and feedback mechanisms in response to targeted therapy, monotherapies are not sufficient to inhibit GBM tumor growth. Unfortunately, much like the disappointing results of the GBM clinical trials using only mTOR inhibitors, the combination of mTOR inhibition with standard chemoradiation yielded poor results [3]. However, preclinical data indicate a synergistic effect of mTOR inhibition with TMZ that may be based on crosstalk between the mTOR pathway and the TMZ-mediated cell death pathway [33,34]. Deciphering the molecular details of this crosstalk may reveal why this therapeutic combination did not translate into an improved clinical outcome. Hence, combining mTOR inhibitors with different drugs targeting other signaling pathways is a much more effective therapeutic approach against GBM, as it improves responses to therapy, overcomes therapy resistance, and addresses GBM heterogeneity. Research on polytherapies for GBM has proposed various combinations, such as pairing mTOR inhibitors with mitogen-activated protein kinase (MEK), cyclin-dependent kinase 4 and 6 (CDK4/6), mouse double minute 2 homolog (MDM2), signal transducer and activator of transcription 3 (STAT3), or growth hormone-releasing hormone (GHRH) inhibitors [35,36,37,38,39]. These and other combination therapies warrant further evaluation to validate which ones are worth positioning for clinical development.

## 7. Conclusions

mTOR represents a hallmark-signaling pathway in GBM and there is a strong rationale for its therapeutic targeting in this aggressive brain tumor. The unexpected results from recent clinical trials of mTOR inhibitors were met with disappointment from the neuro-oncology community. However, rather than abandoning the road for mTOR inhibition in GBM, physicians and researchers in the field should view this outcome as an opportunity to learn, improve, and subsequently pivot to the most promising path forward. As outlined here, it becomes evident that in order to unleash the full potential of mTOR inhibitors in GBM, an integrated approach is required, encompassing improvements in clinical trial design and preclinical studies, a deep understanding of mTOR biology in GBM, the development of more selective mTOR inhibitors, and the identification of the most effective combination therapies based on mTOR inhibitors. Only when all the above are considered and addressed will we arrive at a solid conclusion with respect to the future of mTOR inhibitors in GBM therapy. Results from each front will help direct our next steps on this challenging road of mTOR inhibition in GBM. Most likely, mTOR inhibitors will prove to be effective for a subset of GBM patients with a relevant molecular profile and will eventually be springboarded into the neuro-oncology clinic.

## Figures and Tables

**Table 1:** Examples of novel mTOR inhibitors in GBM clinical trials.

mTOR Inhibitors	Clinical Trials
Dual PI3K/mTOR inhibitors	
NVP-BEZ235	Phase IIB (NCT02430363)
XL765	Phase I/II (NCT01240460)
mTORC1/mTORC2 inhibitors	
INK128	Phase I (NCT02142803)
AZD8055	Phase I (NCT01316809)
AZD2014	Phase I (NCT02619864)
OSI-027	Phase I (NCT00698243)

**Table 2:** Next-generation mTOR inhibitors.

mTOR Inhibitors	Mechanism of Action
Second-generation mTOR inhibitors • Dual PI3K/mTOR inhibitors (PI-103, GNE-477, NVP-BEZ235, BGT226, XL765, SF-1126, and WJD008)	Bind to ATP-binding pocket of both mTOR and PI3K
Second-generation mTOR inhibitors • mTORC1/mTORC2 inhibitors (Torin1, Torin2, PP242, PP30, Ku-0063794, WAY-600, WYE-687, WYE-354, INK128, AZD8055, AZD2014, and OSI-027)	Bind to ATP-binding pocket in the mTOR kinase domain of both mTORC1 and mTORC2
Third-generation mTOR inhibitors RapaLink-1, RapaLink-2	Bind to FKBP12 and ATP-binding pocket of mTORC1 (pharmacophores connected via a linker)
